# Genome-Wide Characterization of Nitrogenase Reductase (*nifH*) Genes in the Sweet Potato [*Ipomoea batatas* (L.) Lam] and Its Wild Ancestors

**DOI:** 10.3390/genes13081428

**Published:** 2022-08-11

**Authors:** Zengzhi Si, Chong Wang, Mingming Zhao, Zhixin Ji, Yake Qiao, Lianjun Wang

**Affiliations:** 1 Hebei Key Laboratory of Crop Stress Biology, Hebei Normal University of Science and Technology, Qinhuangdao 066000, China; 2 Institute of Food Corps, Hubei Academy of Agricultural Sciences, Wuhan 430072, China

**Keywords:** sweet potato, wild ancestors, nitrogenase reductase, phylogenetic analysis, chromosome location, expression profile

## Abstract

The sweet potato (*Ipomoea batatas* (L.) Lam.) is an important and widely grown crop, and the nitrogenase reductase (*nifH*) gene is the most widely sequenced marker gene used to identify nitrogen-fixing bacteria and archaea. There have been many examples of the isolation of the diazotrophic endophytes in sweet potatoes, and there has been no report on whether sweet potatoes and their wild ancestors harbored *nifH* genes. In this study, a comprehensive analysis of *nifH* genes has been conducted on these species by using bioinformatics and molecular biology methods. A total of 20, 19 and 17 *nifH* genes were identified for the first time in sweet potatoes, *I. trifida* and *I. triloba*, respectively. Based on a phylogenetic analysis, all of the *nifH* genes, except for *g10233.t1*, *itf14g14040.t1* and *itb14g15470.t1*, were clustered into five independent clades: I, II, III, IV and V. The *nifH* genes clustered in the same phylogenetic branch showed a more similar distribution of conserved motifs and exons–introns than those of the other ones. All of the identified genes were further mapped on the 15 chromosomes of the sweet potato, *I. trifida* and *I. triloba*. No segmental duplication was detected in each genome of three *Ipomoea* species, and 0, 8 and 7 tandemly duplicated gene pairs were detected in the genome of the sweet potato, *I. trifida* and *I. triloba*, respectively. Synteny analysis between the three *Ipomoea* species revealed that there were 7, 7 and 8 syntenic gene pairs of *nifH* genes detected between the sweet potato and *I. trifida*, between the sweet potato and *I. triloba* and between *I. trifida* and *I. triloba*, respectively. All of the duplicated and syntenic *nifH* genes were subjected to purifying selection inside duplicated genomic elements during speciation, except for the tandemly duplicated gene pair *itf11g07340.t2_itf11g07340.t3*, which was subjected to positive selection. Different expression profiles were detected in the sweet potato, *I. trifida* and *I. triloba*. According to the above results, four *nifH* genes of the sweet potato (*g950*, *g16683*, *g27094* and *g33987*) were selected for quantitative real-time polymerase chain reaction (qRT-PCR) analysis in two sweet potato cultivars (Eshu 15 and Long 9) under nitrogen deficiency (N0) and normal (N1) conditions. All of them were upregulated in the N1 treatment and were consistent with the analysis of the RNA-seq data. We hope that these results will provide new insights into the *nifH* genes in the sweet potato and its wild ancestors and will contribute to the molecular breeding of sweet potatoes in the future.

## 1. Introduction

The sweet potato, *Ipomoea batatas* (L.) Lam., is an important food crop widely grown in the world. It is also an alternative source of bioenergy as a raw material for fuel production [1,2]. The sweet potato contains soluble sugar, starch, dietary fiber, protein, fat, calcium and other minerals, as well as antioxidant substances beneficial to human health, such as carotenoids, anthocyanins, vitamins, flavonoids, etc. [2]. Therefore, the sweet potato is rated as the healthiest vegetable by the World Health Organization.

Nitrogen-fixing microorganisms, as the only natural biological source of fixed nitrogen, play important roles in balancing global ecology [3]. Nitrogen fixation is carried out by nitrogenase, and multiple subunits of nitrogenase are encoded by genes nitrogenase reductase *(nifH)*, alpha subunit *(nifD)* and beta subunit (*nifK*) [4]. Of them, the *nifH* gene, which encodes the nitrogenase reductase subunit, is usually used as the marker gene for studying nitrogen-fixing. Thus, a wide range of environments have been sampled for *nifH* gene diversity [3], such as marine [5], terrestrial [6], extreme [7], anthropogenic [8], host-associated [9] and agricultural [10] environments. Rhizobia is a kind of prokaryotic bacteria with nitrogen fixation abilities [11]. After infecting legumes, it forms many root nodules and converts N_2_ in the air into NH_3_ through root nodules for legumes to use. At the same time, rhizobia can obtain the necessary water and nutrients from root nodule cells [12]. Cyanobacteria, which also plays an important role in the balance of nitrogen elements in nature, is also the pioneer plant in barren lands. Cyanobacteria have a great amount of nitrogen fixation, which also promotes the occurrence of photosynthesis [13]. In addition, *Azolla imbircata* (Roxb.) Nakai, as a floating plant, has the functions of nitrogen fixation, photosynthesis and ammonia release [14,15,16]. The nitrogen fixation of duckweed mainly depends on its photosynthesis, and the energy and reductant required for nitrogen fixation come from photosynthesis [17]. 

The nitrogenase reductase *(nifH)* gene, as the marker gene for studying nitrogen-fixing, has been studied in various fields. Cheng et al. (2018) cloned genes by encoding *nifH*, *nifE*, *nifN* and *nifB* from Heliobacteriumchlorum into the potato virus X (PVX)-basedvector and generated PVX/HisG-*nifH*, PVX/HisG-*nifE*, PVX/HisG-*nifB* and PVX/HisG-*nifN*, and then they inoculated *Nicotiana benthamiana* plants with these recombinant viruses and detected the expression on the translation of all the levels of *nifH*, *nifE*, *nifN* and *nifB* in plants. It was found that *nifH* can be expressed in abundance in plant cells, and the expression of *nifE*, *nifN* and *nifB* are not detectable on SDS-PAGE [18]. Jiang et al. (2021) built a knowledge-based library containing 32 nitrogenase *nifH* sequences from prokaryotes of diverse ecological niches and metabolic features and combined rapid screening in tobacco to identify superior *nifH* variants for plant mitochondria expression. Three *nifH* variants outperformed in tobacco mitochondria and were further tested in yeast [19]. Klarenberg et al. (2022) evaluated the effect of warming and warming-induced shrub expansion on the moss bacterial community composition and diversity, and *nifH* gene abundance and the results showed that the abundance of *nifH* genes was negatively affected by litter abundance [20].

The sweet potato, as a low-nitrogen-tolerant crop, grows well in nitrogen-poor infertile soils. Previous studies have shown that the growth ability of the sweet potato is partially due to the functions of diazotrophic growth-promoting bacteria that contain endo- and epiphytic microorganisms of this plant [21,22,23,24]. Various endophytic bacteria were isolated from sweet potatoes [18], such as *Azospirillum* sp. [25], *Gluconacetobacter* sp. (formerly *Acetobacter* sp.) [26], *Klebsiella* sp. [27], *Pantoea* sp. and *Enterobacter* sp. [28], *Bradyrhizobium* sp., *Paenibacillus* sp., *Pseudomonas* sp. [29], *Enterobacter* sp., *Rahnella* sp., *Rhodanobacter* sp., *Pseudomonas* sp., *Stenotrophomonas* sp., *Xanthomonas* sp., and *Phyllobacterium* sp. [30], etc.

Although there have been many examples of the isolation of the diazotrophic endophytes in sweet potatoes, there has been no report on whether the sweet potato and its wild ancestors harbor *nifH* genes. In this study, a total of 20, 19 and 17 *nifH* genes were first identified in sweet potatoes, *I. trifida* and *I. triloba*, respectively. A phylogenetic analysis grouped these genes into five independent clades: I, II, III, IV and V. Conserved motifs and the gene structures of the *nifH* genes were analyzed. All of the identified genes were further mapped on the 15 chromosomes. Duplication, synteny and Ka/Ks analysis were performed, and the expression profiles of the identified genes were obtained.

## 2. Materials and Methods

### 2.1. Identification and Classification of the nifH Genes

The whole genomes of three *Ipomoea* species, the sweet potato, *I. trifida*, and *I. triloba*, were used in this study. The genome sequences of the sweet potato, including the predicted gene model annotation, were downloaded from the *Ipomoea* Genome Hub (https://ipomoea-genome.org/, accessed on 18 August 2021); the genome sequences of *I. trifida* and *I. triloba*, including the predicted gene model annotation, were downloaded from GenBank BioProject (accessions numbers PRJNA428214 and PRJNA428241). Both a BLAST search and a hidden Markov model search (HMMsearch) were performed as described previously [31]. All protein sequences were first searched for the Fer4_NifH domain (Pfam accession number: PF00142) using hmmsearch with default parameters. Moreover, the extended amino acid sequence of Fer4_NifH domain was used as a query to search for all protein sequences in the sweet potato genome using the BLASTP program. After that, the genes gained by HMMsearch and BLAST methods were merged, and the redundant ones were removed. 

### 2.2. Sequence Alignment and Phylogenetic Analysis

The identified *nifH* genes were aligned using online Clustal Omega (http://www.clustal.org/omega/, accessed on 19 July 2022) [32,33]. According to the previous methods [34], phylogenetic analyses were performed using IQ-TREE (version 1.6.12, http://www.iqtree.org/, accessed on 19 April 2022) with the maximum likelihood algorithm [35], ModelFinder was used to estimate the best-fit model of nucleotide substitution [36], and branch support values were calculated using SH-aLRT and UFBoot2 [37] with 1000 bootstrap replicates [38]. Thus, the obtained tree was summated to Figtree (version 1.4.3) for visual enhancement (http://tree.bio.ed.ac.uk/software/figtree/, accessed on 6 April 2020).

### 2.3. Conserved Motif Detection and Gene Structure Analyses of the nifH Genes

To investigate the structural motif diversity of the identified *nifH* genes, the protein sequences of them were subjected to motif analysis by online MEME SUITE (https://meme-suite.org/meme/, accessed on 19 July 2022) [39]. The criteria used for MEME analysis were (1) a minimum width of 6; (2) a maximum width of 50; (3) a maximum number of motifs designed to identify 20 motifs; and (4) iterative cycles set by default. The exon–intron structure of the *nifH* genes was acquired from the GFF3 annotation files of the sweet potato, *I. trifida* and *I. triloba*. The distribution of conserved motifs and the exon–intron structures of the *nifH* genes were exhibited using TBtools software (version 1.068) (https://github.com/CJ-Chen/TBtools/releases, accessed on 16 July 2021) [40].

### 2.4. Chromosome Distribution of the nifH Genes

The *nifH* genes with chromosome-located positions were mapped on the chromosomes of the sweet potato, *I. trifida* and *I. triloba* using MapChart (version 2.30) software (https://www.wur.nl/en/show/Mapchart.htm, accessed on 3 July 2020) [41]. 

### 2.5. Duplication and Ka/Ks Analysis of the nifH Genes

To search for potential duplicated *nifH* genes in the sweet potato, *I. trifida*, and *I. triloba*, the Multiple Collinearity Scan toolkit (MCScanX, version 0.8) (http://chibba.pgml.uga.edu/mcscan2/, accessed on 3 January 2020) was used [42]. All the *nifH* protein sequences of the three species were compared to themselves by using the BLASTP program with an E-value of 1 × 10^−10^. The resulting blast hits were incorporated along with chromosome coordinates of all *nifH* genes as an input for MCScanX analysis. The hits were classified into various types of duplications, including segmental, tandem, proximal and dispersed under a default criterion. The final results were drawn by CIRCOS software for visualization [43]. The aligned protein sequences of the *nifH* genes of the sweet potato, *I.trifida* and *I.triloba* were first converted into the corresponding nucleotide sequences using PAL2NAL software (http://www.bork.embl.de/pal2nal/#RunP2N, accessed on 25 July 2021) [44] and were then summited to PAML software (version 4.0) (http://abacus.gene.ucl.ac.uk/software/paml.html, accessed on 11 July 2020) [45] for Ka/Ks (nonsynonymous/synonymous) calculation.

### 2.6. Expression Profiles of nifH Genes of the Sweet Potato, I. trifida and I. triloba

For the expression profile analysis of the *nifH* Genes in the sweet potato, *I. trifida* and *I. triloba*, RNA-Seq datasets were downloaded from the sequence read archive (SRA) of NCBI, which referred to different tissues of sweet potatoes (PRJNA511028), and the expressional information (fragments per kilobase of exon model per million mapped fragments, FPKM) of *I. trifida* and *I. triloba* was acquired from the sweet potato Genomics Resource (http://sweetpotato.uga.edu/gt4sp_download.shtml, accessed on 13 December 2021). After removing the low-quality reads and adaptor trimming, the clean RNA-Seq reads were aligned to the genome sequences of the sweet potato via Hisat2 [46]. Thereafter, SAMtools software (version 1.11) was used for aligned read counting (https://github.com/samtools/samtools/releases/download/1.11, accessed on 23 December 2020) [47]. Then, the obtained read counts were imported into DEseq2 for the analysis of differentially expressed genes (DEGs) [46]. For each compared course, it was treated as a DEG if |log2FC| > 1 and FDR ≤ 5%, and a mean log2FC value for each gene was calculated. The heat map was produced to distribute the expression levels using the RPKM (i.e., reads per kilobase per million) value in MeV software (version 4.9.0) (https://sourceforge.net/projects/mev/, accessed on 23 August 2020) [48].

### 2.7. RNA Isolation and Quantitative Real-Time Polymerase Chain Reaction (qRT-PCR) Analysis

Two sweet potato cultivars (Eshu 15, medium long vine; Long 9, short vine) were selected for the qRT-PCR analysis of the *nifH* genes. Freshly cut sweet potato seedlings, with 20- to 30-cm-long stems and five to seven leaves, were dipped into water for transplanting for 3 days. Then, the seedlings were transferred into a Hoagland nutrient solution, and the nutrient solution was set with the following nitrogen levels: N0 (0 mmol·L^−1^ pure nitrogen) and N1 (14 mmol·L^−1^ pure nitrogen). Other components remained the same: 1 mmol·L^−^^1^ KH_2_PO_4_, 2 mmol·L^−^^1^ MgSO_4_, 2.50 mmol·L^−^^1^ K_2_SO_4_, 20 mmol·L^−^^1^ FeSO_4_·7H_2_O, 20 mmol·L^−^^1^ EDTA-Na_2_·2H_2_O, 5 μmol·L^−^^1^ NaI, 0.10 mmol·L^−^^1^ H_3_BO_3_, 0.15 mmol·L^−^^1^ MnSO_4_, 0.05 mmol·L^−^^1^ ZnSO_4_, 1 μmol·L^−^^1^ (NH_4_)_2_MoO_4_, 0.16 μmol·L^−^^1^ CuSO_4_ and 0.19 μmol·L^−1^ CoCl_2_. Leaf samples were then collected at three time points: 0 h, 3 h and 72 h after treatment. Thereafter, the total RNA of the samples was isolated using the FastPure ^®^ Universal Plant Total RNA Isolation Kit (TransGen, Wuhan, China), and the first-strand cDNA was prepared using EasyScript All-in-One First-Strand cDNA Synthesis SuperMix for qPCR (One-Step gDNA Removal) (TransGen, Wuhan, China). The sweet potato β-actin gene (Genbank AY905538) was selected and used to normalize the relative quantities of the target genes. Three replications were performed, and the expression changes were calculated using the 2^–ΔΔCt^ method for each sample. Then, a quantitative real-time polymerase chain reaction (qRT-PCR) for four sweet potato *nifH* genes (*g950*, *g16683*, *g27094* and *g33987*) was performed. The primers used for PCR were designed using on line Primer-BLAST (http://www.ncbi.nlm.nih.gov/tools/primer-blast/, accessed on 21 July 2022) (Table S1). 

## 3. Results

### 3.1. Identification of the nifH Genes of the Sweet Potato, I. trifida and I. triloba

A total of 20, 19 and 17 *nifH* genes were identified in the genomes of the sweet potato, *I. trifida* and *I. triloba*, respectively (Table 1). Among the 20 *nifH* genes in the sweet potato, the shortest (*g56705.t1*) was of 67 amino acids, whereas the longest (*g4650.t1*) was of 939 amino acids, and the average length of the genes was 430.55 amino acids (Table 1). Among the 19 *nifH* genes in *I. trifida*, the shortest (*itf12g22980.t1*) was of 289 amino acids, whereas the longest (*itf12g19260.t1*) was of 530 amino acids, and the average length of the genes was 372.21 amino acids (Table 1). Among the 17 *nifH* genes in *I. triloba*, the shortest (*itb11g03920.t3*) was of 278 amino acids, whereas the longest (*itb14g15470.t1*) was of 611 amino acids, and the average length of the genes was 385 amino acids (Table 1).

### 3.2. Phylogenetic Analysis of the nifH Genes of the Sweet Potato, I. trifida and I. triloba

**Figure 1:**
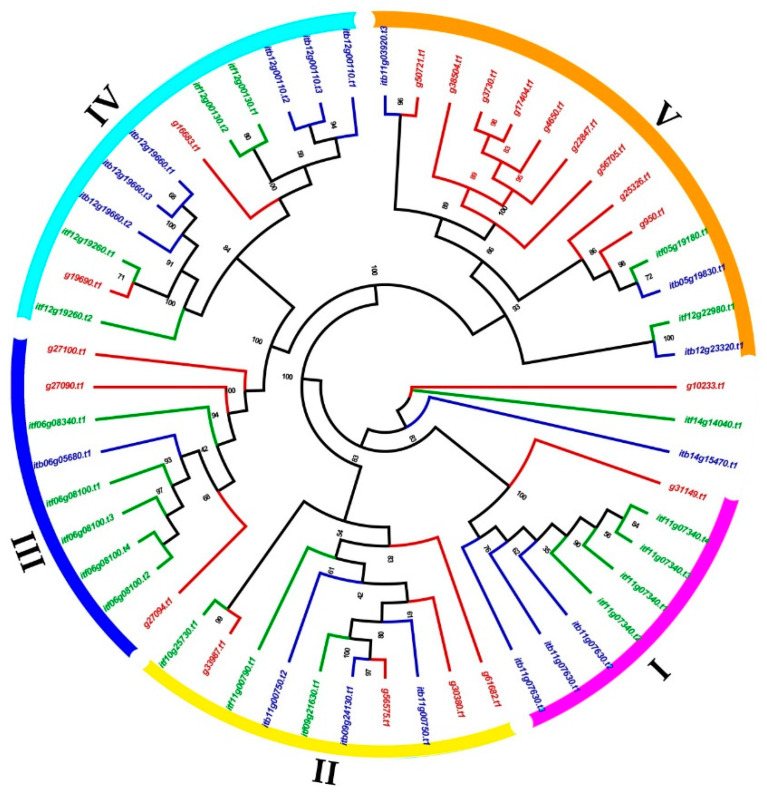
Phylogenetic relationships of the *nifH* genes in sweet potato, *I. trifida* and *I. triloba* based on the amino acids. Red, green and blue lines represent the *nifH* genes in sweet potato, *I. trifida*, and *I. triloba*, respectively.

To analyze the phylogenetic relationship of the *nifH* genes in the sweet potato, *I. trifida* and *I. triloba*, a phylogenetic tree was constructed (Figure 1). All of the *nifH* genes, except for *g10233.t1*, *itf14g14040.t1* and *itb14g15470.t1*, were clustered into five independent clades: I, II, III, IV and V, with support values > 93% (Figure 1). Each of the five clades contained the *nifH* genes from all of the three species, which indicated that the ancestries for each clade were differentiated before the species specification of the *nifH* genes. Among the genes that were not clustered, the same phenomenon was detected; *g10233.t1*, *itf14g14040.t1* and *itb14g15470.t1* were identified from the genome of the sweet potato, *I. trifida* and *I. triloba*, respectively (Figure 1). Although the number of the *nifH* genes in the sweet potato, *I. trifida* and *I. triloba* for each clade was different, it meant that the *nifH* genes of each clade may have experienced different duplication during the species specification of the *nifH* genes (Figure 1). 

### 3.3. Conserved Motif Detection and Gene Structure Analyses of the nifH Genes

**Figure 2:**
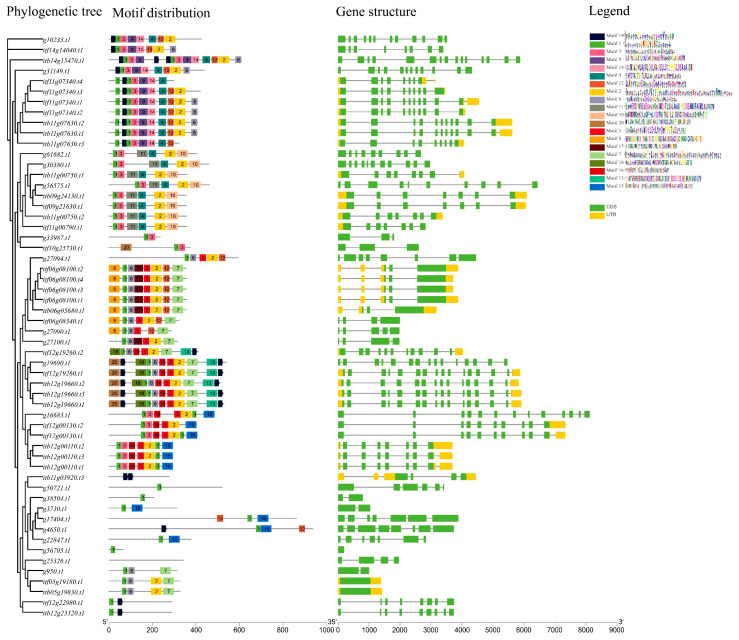
Conserved motifs and gene structure distribution of the *nifH* genes.

Conserved motifs were detected in all of the identified *nifH* genes except for *g25326.t1* in the three *Ipomoea* species (Figure 2). Among these conserved motifs, motif 1 was the most conserved one, and 54 of the 56 *nifH* genes harbored motif 1, followed by motif 2 (39 of 56), motif 3(27 of 56) and motif 6 (25 of 56). Of the identified *nifH* genes, *itb14g15470.t1* contained the greatest number of conserved motifs (#14), followed by *g19690.t1* (#11), *itf12g19260.t1* (#11), *itb12g19660.t2* (#11), *itb12g19660.t3* (#11), *itb12g19660.t1* (#11), *itf11g07340.t1* (#10), *itf11g07340.t2* (#10), *itb11g07630.t2* (#10) and *itb11g07630.t1* (#10). It was also found that the *nifH* genes clustered in the same phylogenetic branch showed a more similar distribution of conserved motifs and exons–introns (gene structure) than those of the other *nifH* genes (Figure 2).

### 3.4. Chromosome Locations of the nifH Genes of the Sweet Potato, I. trifida and I. triloba

**Figure 3:**
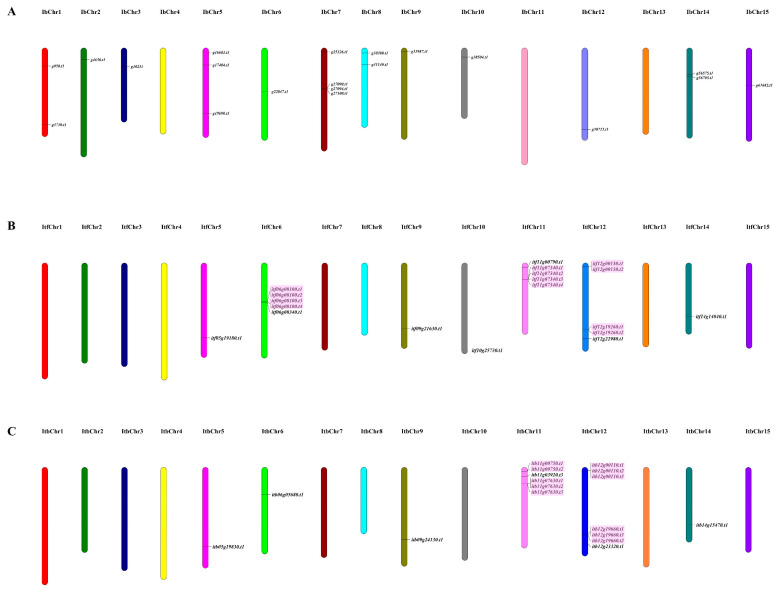
**Distribution of *nifH* genes in chromosomes:** (**A**) Distribution in *I. batatas* chromosomes. (**B**) Distribution in *I. trifida* chromosomes. (**C**) Distribution in *I. triloba* chromosomes. Randomly replicated *nifH* genes are shown with a pink background.

Based on the locations of individual *nifH* genes, all of the identified genes were mapped on the 15 chromosomes of the sweet potato, *I. trifida* and *I. triloba*, respectively (Figure 3). However, the chromosome distribution of the *nifH* genes was similar between *I. trifida* and *I. triloba*, whereas it was different between the sweet potato and *I. trifida* or *I. triloba* (Figure 3). The *nifH* genes in the sweet potato were distributed on all of the chromosomes except for chromosome 4, 11 and 13, whereas the *nifH* genes in *I. trifida* and *I. triloba* were mainly located on chromosome 5, 6, 9, 11, 12 and 14 (Figure 3). 

### 3.5. Duplication and Ka/Ks Analysis of the nifH Genes

**Figure 4:**
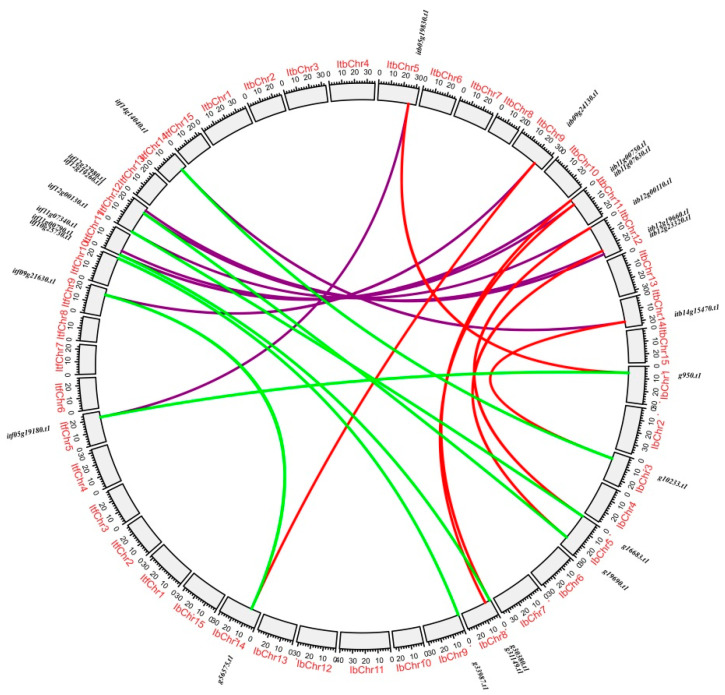
**Synteny analysis of the *nifH* genes.** The outer circle represents the haploid chromosomes of the sweet potato, *I. trifida* and *I. triloba* (gray); red, green and purple lines show the syntenic gene pairs between sweet potato and *I. trifida*, between sweet potato and *I. triloba* and between *I. trifida* and *I. triloba*, respectively.

The duplication analysis of the *nifH* genes in the three *Ipomoea* species showed that no segmental duplication was detected in each genome of them, and 0, 8 and 7 tandemly duplicated gene pairs were detected in the genome of the sweet potato, *I. trifida* and *I. triloba*, respectively (Figure 3). The synteny analysis between the three *Ipomoea* species revealed that there were 7, 7 and 8 syntenic gene pairs of *nifH* genes detected between the sweet potato and *I. trifida*, between the sweet potato and *I. triloba* and between *I. trifida* and *I. triloba*, respectively (Figure 4).

The non-synonymous substitution (Ka) to synonymous substitution (Ks) ratio (Ka/Ks) is an informative value of positive selection. To detect whether some *nifH* genes are under positive selection, Ka/Ks analysis was performed on duplicated and syntenic *nifH* genes within or between the studied three *Ipomoea* species. All of the duplicated and syntenic gene pairs showed a Ka/Ks ratio <1, except for the tandemly duplicated gene pair *itf11g07340.t2_itf11g07340.t3*, which has a Ka/Ks ratio = 1.34 (Table 2). The results revealed that *itf11g07340.t2_itf11g07340.t3* was subjected to positive selection, and all of the other duplicated and syntenic *nifH* genes were subjected to purifying selection inside duplicated genomic elements during speciation.

### 3.6. Expression Patterns of the nifH Genes in the Sweet Potato, I. trifida and I. triloba

**Figure 5:**
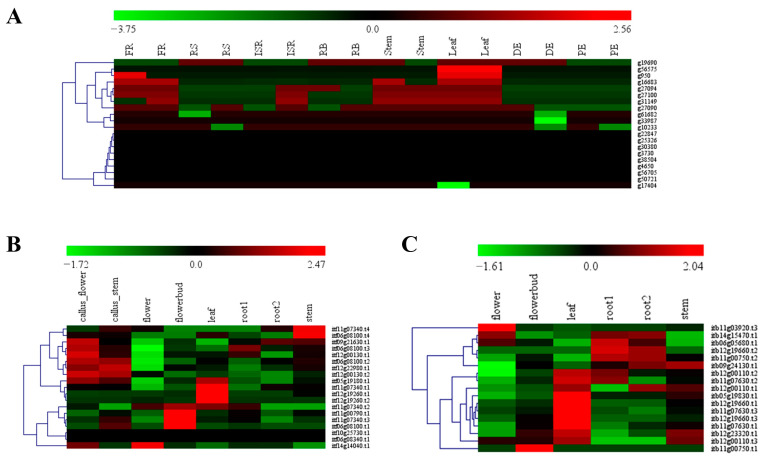
**Heatmaps of expression profiles for *nifH* genes in multiple tissues:** (**A**) Expression profiles of *nifH* genes in different sweet potato tissues: fibrous roots (FR), initiative storage roots (ISR), leaves, distal ends (DE), proximal ends (PE), root bodies (RB) and root stalks (RS); (**B**) Expression profiles of *nifH* genes in different *I. trifida* tissues: callus flowers, callus stems, flowers, flower buds, root1, root2, leaves and stems; (**C**) Expression profiles of *nifH* genes in different *I. triloba* tissues: root1, root2, flowers, flower buds, leaves and stems.

In the sweet potato, nearly half of the identified *nifH* genes (9 genes) were nonregulated in all of detected tissues. The others were mainly upregulated in the fibrous roots (FR) and leaves and were mainly downregulated in the proximal ends (PE) and root stalks (RS) (Figure 5). In the *I. trifida*, 8, 6, 1, 4, 4 and 2 of the 19 identified *nifH* genes were upregulated in the callus flowers, callus stems, flowers, flower buds, leaves and stems, respectively, and other genes in the tissue mentioned above or the 19 identified *nifH* genes in other tissues (i.e., root1 and root2) were mainly downregulated (Figure 5). In the *I. triloba*, 3, 1, 10, 5 and 4 of the 17 identified *nifH* genes were upregulated in the flowers, flower buds, leaves, root1, root2 and stems, respectively, whereas other genes in the tissue mentioned above were mainly downregulated (Figure 5). 

### 3.7. qRT-PCR Analysis of the nifH Genes under Treatments

**Figure 6:**
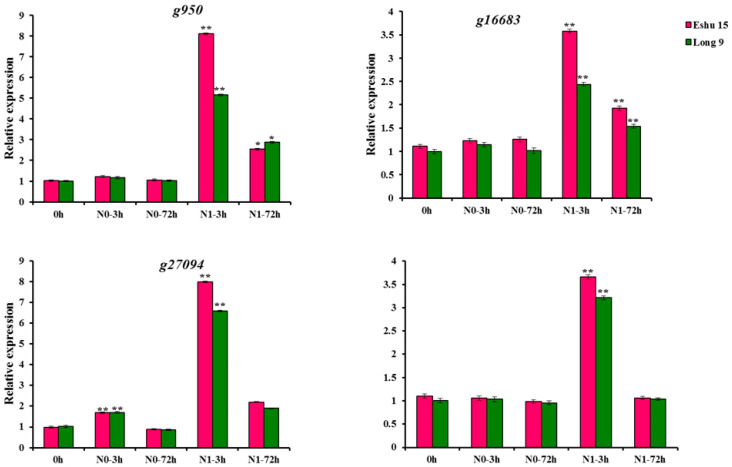
**Expression analysis of *g950*, *g16683*, *g27094* and *g33987* of Eshu 15 and Long 9.** The significance of differential gene expression levels compared with control are denoted as * < 0.05, ** < 0.01.

According to the above results, four *nifH* genes of the sweet potato (*g950*, *g16683*, *g27094* and *g33987*) were selected for qRT-PCR analysis. All of the four genes were upregulated in N1 treatment and were consistent with the above analysis of RNA-seq data (Figure 6). Compared with the control conditions (0 h), the transcripts of the four *nifH* genes all peaked after 3 h N1 treatment in the two sweet potato culitivars. Then, the expression level declined, and the expression level of Eshu 15 was higher than that of Long 9 (Figure 6). In Eshu 15, *g950*, peaked at 3 h with an 8.11 fold higher expression level than that of the control, which was of the highest expression levels of the four *nifH* genes, followed by *g27094* (7.99 fold), *g33987* (3.66 fold) and *g16683* (3.58 fold). In Long 9, *g27094*, peaked at 3 h with a 6.60 fold higher expression level than that of the control, which was of the highest expression levels of the four *nifH* genes, followed by *g950* (5.16 fold), *g33987* (3.21 fold) and *g16683* (2.44 fold) (Figure 6). However, in the N0 treatment, the transcripts of *g950, g16683* and *g33987* had all almost no change compared with the control conditions (0 h), and the transcripts of *g27094* peaked after 3 h N0 treatment, with expression levels of only 1.69 and 1.69 fold higher than that of the control in Eshu 15 and Long 9, respectively (Figure 6).

## 4. Discussion

Nitrogen is an essential element for plants, and it is also one of the most important limiting factors for obtaining a high agricultural yield. Biological nitrogen fixation is an important part of the terrestrial nitrogen cycle, which contributes 90–130 Tg N to the biosphere every year and is mainly completed by bacteria and leguminous plants [49]. *nifH* is a marker gene, and researchers have been able to characterize aspects of the diversity and ecology of nitrogen-fixing bacteria and archaea. The biological nitrogen fixation of plants, rather than by association with microorganisms, can generate crops that are less dependent on synthetic nitrogen fertilizers and can increase agricultural productivity and sustainability [50]. The sweet potato, the seventh largest food crop in the world [2], grows well in nitrogen-poor infertile soils, and it is believed that sweet potato endo- and epiphytic microorganisms play important roles in acting upon this growth ability in this plant [21,22,23,24]. Are these all the reasons? Do the sweet potato and its wild ancestors harbor *nifH* genes, even with nitrogen fixation potentiality?

Previous studies have demonstrated that, among the 291 tested accessions of cultivated sweet potatoes, all contained one or more transfer DNA (T-DNA) sequences, which is believed to be harbored by *Agrobacterium* [51]. The study suggested that an agrobacterium infection occurred in evolutionary times, and the T-DNA integration, the interruption of an F-box gene and the subsequent fixation of foreign T-DNA into the sweet potato genome occurred during the evolution and domestication of the sweet potato [51]. Based on the above demonstration, the sweet potato and its wild ancestors harboring *nifH* genes is possible.

In order to answer the question mentioned above, in this present study, the genome-wide identification of *nifH* genes was conducted for the first time in this study. A total of 20, 19 and 17 *nifH* genes were identified in the genome of the sweet potato, *I. trifida* and *I. triloba*, respectively. The number of *nifH* genes in the three investigated species was comparable. The phylogenetic analysis revealed that the identified *nifH* genes can be clustered into five independent clades: I, II, III, IV and V, with high support values. Moreover, each of the five clades contained the *nifH* genes from all of the three species, which indicated that the ancestries for each clade were differentiated before the species specification of the *nifH* genes. All of the *nifH* genes could be located on chromosomes of the three species, and the distribution of them on sweet potatoes were different from *I.trifida* and *I.triloba*. Previous reports have suggested that the whole-genome triplication (WGT) occurred in an ancient ancestor of the *Ipomoea* lineage around 46.1 million years ago (Mya), much earlier than the divergence of *I. nil* from the lineage containing *I. trifida* and *I. triloba* (~3.6 Mya) and the *I. trifida*-*I. triloba* divergence (~2.2 Mya). The results of the comparison between the genomes of *I. trifida* (or *I. triloba*) and I. nil limited large-scale interchromosomal rearrangements over the last 3.6 million years [52], and in the sweet potato, two recent whole-genome duplication (WGD) events occurred about 0.8 and 0.5 million years ago [53]. Therefore, the two recent whole-genome duplications in the sweet potato may be the reason for the discrepancies of chromosome distribution.

Conserved motif detection and gene structure analyses showed that the detected motifs behaved with different degrees of conservation among the *nifH* genes, and the *nifH* genes clustered in the same phylogenetic branch showed a more similar distribution of conserved motifs and exons–introns than that of the other *nifH* genes. Similar results have been found in other gene families in various species, such as the WRKY gene family in pineapples [54], the AP2/ERF gene family in buckwheat [55], the superoxide dismutase (SOD) gene family in rapeseed [56], etc.

Segmental and tandem duplications have significantly contributed to gene family expansion in plants [57,58]. The duplication analysis in the present study shows that no segmental duplication was found in each genome of the three *Ipomoea* species, and 0, 8 and 7 tandemly duplicated gene pairs were detected in the genome of the sweet potato, *I. trifida* and *I. triloba*, respectively. The results suggest that there were no segmental duplications of *nifH* genes through polyploidy followed by chromosome rearrangements, and in several members of them in *I. trifida* and *I. triloba*, there occurred duplications within the same intergenic region or in neighboring intergenic regions of their genomes [59]. A total of 7, 7 and 8 syntenic gene pairs of *nifH* genes were detected between the sweet potato and *I. trifida*, between the sweet potato and *I. triloba* and between *I. trifida* and *I. triloba*, respectively. Similar results have been demonstrated by other closely related species. For example, in the Brassicaceae family, a number of NBS loci were identified: *Arabidopsis lyrata* (#78), *A. thaliana* (#58), *Brassica rapa* (#100), *Capsella rubella* (#52) and *Thellungiella salsuginea* (#59) [57]. These may be generated by different extents of the gene duplication of the ancestor genes [60]. The Ka/Ks analysis of the duplicated and syntenic *nifH* genes in the three *Ipomoea* species revealed that nearly all of the duplicated and syntenic *nifH* genes were subjected to purifying selection inside duplicated genomic elements during speciation, since they had a ratio of Ka/Ks < 1 [61].

The expression profile of the *nifH* genes showed that all but nine of the sweet potato genes detected upregulation or downregulation in various tissues of their corresponding species. Four *nifH* genes of the sweet potato were selected for qRT-PCR analysis, and the expressions were consistent with the transcriptome data analysis. It was also found that the *nifH* genes expressed differently in the three species, and the *nifH* genes expressed differently in the different tissues of the same species as well. The results suggest that the *nifH* genes experienced functional differentiation after the whole genome duplication. The phenomenon of the member of the family gene acting differently in expression profiles has been reported in various studies, for example, in wheat [62], tomatoes [63], tea [64], etc., and even in the duplicated genes [65]. In potatoes, it was found that there was variation within tandemly duplicated genes among cultivated, non-cultivated and wild potato genotypes in terms of bias in functional specificities, the proportion of lineage-specific clusters, diverged expression and promoter similarities [66].

## 5. Conclusions

In this study, A total of 20, 19 and 17 *nifH* genes were identified for the first time in the sweet potato, *I.trifida* and *I.triloba*, respectively. Following the identification, a phylogenetic tree was formed to cluster the identified *nifH* genes into five independent clades. All of the *nifH* genes could be located on chromosomes of the three species, and the distribution of them on sweet potatoes were different from *I.trifida* and *I.triloba*. The expression profiles revealed that the *nifH* genes were expressed differently in various tissues of the three species. A total of 4 *nifH* genes of the sweet potato were selected for qRT-PCR analysis in two sweet potato cultivars (Eshu 15 and Long 9) under nitrogen deficiency (N0) and normal (N1) conditions. The results acquired in this study may provide new insight into the *nifH* genes in the sweet potato and its wild ancestors, and they may contribute to the nitrogen-fixing breeding of sweet potatoes in the future.

## Figures and Tables

**Table 1:** The *nifH* gene information of sweet potato, *I. trifida* and *I. triloba*.

Species	Gene_Id	Number of Amino Acids	Exon Number	Chromosome Location	Strand	Gene_Start	Gene_End
	*g950.t1*	315	2	Ibchr1	+	5,540,056	5,541,373
	*g3730.t1*	315	3	Ibchr1	+	26,923,828	26,934,096
	*g4650.t1*	939	8	Ibchr2	+	3,249,593	3,253,657
	*g10233.t1*	426	13	Ibchr3	+	5,697,280	5,701,545
	*g16683.t1*	488	13	Ibchr5	−	550,499	558,988
	*g17404.t1*	864	8	Ibchr5	+	5,150,173	5,157,672
	*g19690.t1*	541	16	Ibchr5	+	22,748,809	22,759,672
	*g22847.t1*	378	8	Ibchr6	−	14,879,621	14,883,661
	*g25326.t1*	343	4	Ibchr7	−	363,695	365,842
sweet potato	*g27090.t1*	289	6	Ibchr7	+	13,784,034	13,787,412
	*g27094.t1*	594	8	Ibchr7	+	13,802,034	13,807,229
	*g27100.t1*	317	4	Ibchr7	+	13,837,054	13,839,787
	*g30380.t1*	460	11	Ibchr8	−	761,393	764,513
	*g31149.t1*	440	12	Ibchr8	−	4,991,243	4,995,748
	*g33987.t1*	237	3	Ibchr9	+	217,387	219,435
	*g38504.t1*	207	3	Ibchr10	−	2,286,298	2,293,174
	*g50721.t1*	522	7	Ibchr12	−	28,604,621	28,610,924
	*g56575.t1*	464	10	Ibchr14	−	8,492,679	8,500,020
	*g56705.t1*	67	2	Ibchr14	+	9,444,490	9,445,508
	*g61682.t1*	405	9	Ibchr15	+	12,526,772	12,530,022
	*itf05g19180.t1*	329	1	Chr05	−	21,097,894	21,099,282
	*itf06g08100.t1*	358	5	Chr06	−	10,472,405	10,476,280
	*itf06g08100.t2*	358	5	Chr06	−	10,472,405	10,476,280
	*itf06g08100.t3*	358	5	Chr06	−	10,472,558	10,476,272
	*itf06g08100.t4*	358	5	Chr06	−	10,472,558	10,476,272
	*itf06g08340.t1*	323	4	Chr06	+	10,684,647	10,686,646
	*itf09g21630.t1*	357	7	Chr09	+	18,324,833	18,330,898
	*itf10g25730.t1*	377	3	Chr10	−	24,717,023	24,719,626
	*itf11g00790.t1*	359	7	Chr11	−	370,700	373,523
*I. trifida*	*itf11g07340.t1*	410	12	Chr11	−	3,956,871	3,961,420
	*itf11g07340.t2*	410	11	Chr11	−	3,957,323	3,961,420
	*itf11g07340.t3*	421	10	Chr11	−	3,957,967	3,961,420
	*itf11g07340.t4*	299	9	Chr11	−	3,958,281	3,961,420
	*itf12g00130.t1*	409	11	Chr12	−	131,709	139,045
	*itf12g00130.t2*	405	10	Chr12	−	131,709	139,045
	*itf12g19260.t1*	530	16	Chr12	+	18,656,207	18,662,081
	*itf12g19260.t2*	411	12	Chr12	+	18,658,050	18,662,081
	*itf12g22980.t1*	289	8	Chr12	−	21,330,987	21,334,731
	*itf14g14040.t1*	311	8	Chr14	−	14,807,579	14,810,974
	*itb05g19830.t1*	329	1	Chr05	−	26,142,356	26,143,775
	*itb06g05680.t1*	358	4	Chr06	−	8,346,489	8,349,665
	*itb09g24130.t1*	357	7	Chr09	+	23,745,236	23,751,328
	*itb11g00750.t1*	361	8	Chr11	−	336,194	340,261
	*itb11g00750.t2*	359	7	Chr11	−	336,931	340,298
	*itb11g03920.t3*	278	6	Chr11	+	2,113,511	2,117,952
	*itb11g07630.t1*	408	12	Chr11	−	4,684,828	4,690,449
	*itb11g07630.t2*	408	11	Chr11	−	4,684,828	4,690,449
*I. triloba*	*itb11g07630.t3*	324	9	Chr11	−	4,686,363	4,690,421
	*itb12g00110.t1*	297	9	Chr12	−	146,125	149,815
	*itb12g00110.t2*	297	8	Chr12	−	146,125	149,815
	*itb12g00110.t3*	297	9	Chr12	−	146,125	149,815
	*itb12g19660.t1*	530	16	Chr12	+	22,066,146	22,072,061
	*itb12g19660.t2*	513	15	Chr12	+	22,066,170	22,072,003
	*itb12g19660.t3*	529	16	Chr12	+	22,066,146	22,072,061
	*itb12g23320.t1*	289	9	Chr12	−	25,103,363	25,107,101
	*itb14g15470.t1*	611	15	Chr14	−	18,766,811	18,772,688

**Table 2:** Ka/Ks analysis of the duplicated and syntenic *nifH* genes in and between the three *Ipomoea* species.

Gene Pairs	Duplication Type	Ka	Ks	Ka/Ks
*g10233.t1_itb14g15470.t1*	Segmental replication	0.06	0.11	0.54
*g10233.t1_itf14g14040.t1*	Segmental replication	0.05	0.12	0.39
*g16683.t1_itb12g00110.t1*	Segmental replication	0.00	0.00	0.62
*g16683.t1_itf12g00130.t1*	Segmental replication	0.00	0.00	0.65
*g19690.t1_itb12g19660.t1*	Segmental replication	0.01	0.03	0.21
*g19690.t1_itf12g19260.t1*	Segmental replication	0.00	0.03	0.00
*g30380.t1_itb11g00750.t1*	Segmental replication	0.00	0.05	0.02
*g30380.t1_itf11g00790.t1*	Segmental replication	0.00	0.02	0.00
*g31149.t1_itb11g07630.t1*	Segmental replication	0.01	0.05	0.16
*g33987.t1_itf10g25730.t1*	Segmental replication	0.11	0.13	0.82
*g56575.t1_itb09g24130.t1*	Segmental replication	0.02	0.07	0.25
*g56575.t1_itf09g21630.t1*	Segmental replication	0.02	0.08	0.26
*g950.t1_itb05g19830.t1*	Segmental replication	0.00	0.05	0.06
*g950.t1_itf05g19180.t1*	Segmental replication	0.00	0.07	0.04
*itb05g19830.t1_itf05g19180.t1*	Segmental replication	0.00	0.06	0.00
*itb09g24130.t1_itf09g21630.t1*	Segmental replication	0.00	0.03	0.08
*itb11g00750.t1_itf11g00790.t1*	Segmental replication	0.00	0.06	0.02
*itb11g07630.t1_itf11g07340.t1*	Segmental replication	0.00	0.04	0.03
*itb12g00110.t1_itf12g00130.t1*	Segmental replication	0.00	0.01	0.15
*itb12g19660.t1_itf12g19260.t1*	Segmental replication	0.01	0.04	0.14
*itb12g23320.t1_itf12g22980.t1*	Segmental replication	0.00	0.02	0.00
*itb14g15470.t1_itf14g14040.t1*	Segmental replication	0.02	0.07	0.32
*itf11g07340.t2_itf11g07340.t3*	Tandem	0.08	0.06	1.34
*itf11g07340.t3_itf11g07340.t4*	Tandem	0.03	0.04	0.71
*itf12g19260.t1_itf12g19260.t2*	Tandem	0.02	0.02	0.62
*itb11g00750.t1_itb11g00750.t2*	Tandem	0.00	0.00	0.30
*itb11g07630.t2_itb11g07630.t3*	Tandem	0.01	0.01	0.59

## Data Availability

The genome sequences of the sweet potato, including the predicted gene model annotation for this study, can be found in the *Ipomoea* Genome Hub (https://ipomoea-genome.org/, accessed on 18 August 2021); the genome sequences of *I. trifida* and *I. triloba*, including the predicted gene model annotation, can be found in GenBank BioProject (accessions numbers PRJNA428214 and PRJNA428241). The RNA-Seq datasets referring to different tissues of the sweet potato and I. nil can be acquired from SRA of NCBI (PRJNA511028); the expressional information (fragments per kilobase of exon model per million mapped fragments, FPKM) of *I. trifida* and *I. triloba* can be obtained from the sweet potato Genomics Resource (http://sweetpotato.uga.edu/gt4sp_download.shtml, accessed on 13 December 2021).

## Supplementary Materials

The following supporting information can be downloaded at: https://www.mdpi.com/article/10.3390/genes13081428/s1, Table S1: Primers used in qRT-PCR.
